# Toward a More Usable Home-Based Video Telemedicine System: A Heuristic Evaluation of the Clinician User Interfaces of Home-Based Video Telemedicine Systems

**DOI:** 10.2196/humanfactors.7293

**Published:** 2017-04-24

**Authors:** Sruthy Agnisarman, Shraddhaa Narasimha, Kapil Chalil Madathil, Brandon Welch, FNU Brinda, Aparna Ashok, James McElligott

**Affiliations:** ^1^ Department of Civil Engineering Clemson University Clemson, SC United States; ^2^ Department of Industrial Engineering Clemson University Clemson, SC United States; ^3^ MUSC Center for Telehealth Department of Public Health Sciences Medical University of South Carolina Charleston, SC United States; ^4^ School of Dental Medicine Southern Illinois University at Edwardsville Edwardsville, IL United States

**Keywords:** telemedicine, heuristics, Internet, user-computer interface, cognition

## Abstract

**Background:**

Telemedicine is the use of technology to provide and support health care when distance separates the clinical service and the patient. Home-based telemedicine systems involve the use of such technology for medical support and care connecting the patient from the comfort of their homes with the clinician. In order for such a system to be used extensively, it is necessary to understand not only the issues faced by the patients in using them but also the clinician.

**Objectives:**

The aim of this study was to conduct a heuristic evaluation of 4 telemedicine software platforms—Doxy.me, Polycom, Vidyo, and VSee—to assess possible problems and limitations that could affect the usability of the system from the clinician’s perspective.

**Methods:**

It was found that 5 experts individually evaluated all four systems using Nielsen’s list of heuristics, classifying the issues based on a severity rating scale.

**Results:**

A total of 46 unique problems were identified by the experts. The heuristics most frequently violated were *visibility of system status* and *Error prevention* amounting to 24% (11/46 issues) each. *Esthetic and minimalist design* was second contributing to 13% (6/46 issues) of the total errors.

**Conclusions:**

Heuristic evaluation coupled with a severity rating scale was found to be an effective method for identifying problems with the systems. Prioritization of these problems based on the rating provides a good starting point for resolving the issues affecting these platforms. There is a need for better transparency and a more streamlined approach for how physicians use telemedicine systems. Visibility of the system status and speaking the users’ language are keys for achieving this.

## Introduction

### Health Care System

The health care system in the United States is currently undergoing extensive changes. Possible causes for such challenges faced by its delivery system are increased demand for health care due to an increased number and changing lifestyle leading to an increase in chronic diseases, the demand for increased accessibility of care outside hospitals, moving health services into the patient’s own homes, the need for increased efficiency, individualization and equity of quality-oriented health care with limited financial resources, the difficulties of recruiting and retaining personnel in health care services in general, and in home and elderly care in particular [1,2]. Telehealth, the use of electronic information and telecommunication technologies to support long-distance clinical health care, patient and professional health-related education, public health and health administration [1,3], has the potential to address these issues. One subsection, telemedicine, the use of technology to provide and support health care when distance separates the clinical service and the patient, appears to be particularly attractive [4].

Although playing an important role in addressing the health issues of patients living in underserved and rural areas, telemedicine is now attracting attention beyond these limited regions. It offers mechanisms for centralizing specialists, reducing costs for specialty care, and supporting primary care clinician needs in the urban and suburban areas these typically serve [5-9]. The possible benefits of using these systems have resulted in an increased interest in telemedicine. For example, they help patients with chronic illness and those with limited mobility to connect with a health care facility from the comfort of their homes [1], and it is important because it reduces the stress for patients who otherwise would have to travel long distances for their appointments [10]. Currently, this remote care is extensively used for clinical visits that do not require physical presence such as behavioral health [11,12], counseling [13-15], follow-up [8,16], and patient education [17], with studies finding telemedicine an appealing solution for the real-time remote monitoring of patients. It has also shown promise for improving patient knowledge of health care, thus helping them better manage their diseases or illnesses. With the expanding technical capabilities and the decreasing costs of telemedicine software solutions, home-based telemedicine is becoming more widely used, evidenced by a recent workshop conducted by the National Academies that discussed the potential of scaling such delivery of care for a growing number of patients [2,18].

### Telemedicine System

Unlike face-to-face encounters, in which clinicians and patients are both located in the same setting, telemedicine participants usually use the teleconferencing systems at their respective locations. Thus, for a telemedicine system to become widely accepted, it should not only be functional but also user-friendly [19-23]. The Institute of Medicine (IOM) recently emphasized the role of usability in telemedicine systems, given its potential to replace regular clinical visits which are both time-consuming and resource-demanding [2,8,23].

However, limited research has been conducted evaluating the perceived usefulness and usability of such tools from a home-based video telemedicine system perspective [24,25]. The evaluation of a user interface can be carried out by 4 ways; *formally* using analytical tools, *automatically* using computer technology, *empirically*, that is, testing with users, and *heuristically* [25-27]. Heuristic evaluation is the process of usability testing wherein evaluators are provided with an interface and asked to comment on it based on a set of heuristics [27,28]. The efficiency of this system of evaluation allows for an iterative design process of user interfaces [18]. Studies have found that this type of evaluation can reveal both major and minor usability issues, including problems that lead to errors and user dissatisfaction content [29-31]. Furthermore, it has been extensively used to ascertain usability issues in the medical field ranging from websites to medical devices [25,32-34] to health information technology applications [35-40]. In light of these advantages of a heuristic evaluation, this study aimed to understand the issues of the clinician’s interfaces of 4 telemedicine platforms. The issues uncovered through this heuristic evaluation could serve as a basis to improve the clinician’s interface in telemedicine platforms.

## Methods

### Telemedicine Systems

The criteria for a telemedicine system to be included in this heuristic evaluation were as follows: (1) the system is primarily used to deliver video-based telemedicine at home; (2) the system does not require specialized or proprietary equipment for home use; (3) the system runs on an Internet-connected computer with audio and video capabilities; and (4) the system is Health Insurance Portability and Accountability Act (HIPAA) compliant, which aims at protecting the health rights and privacy of patiens [41]. Initially, the telemedicine systems used by the medical staff at the Medical University of South Carolina (MUSC) Center for Telehealth and South Carolina (SC) Telehealth Alliance were reviewed. Subsequently, 8 software applications, Adobe Connect, Cisco WebEx, Cisco Jabber, Doxy.me, Polycom, Skype, Vidyo, and VSee, were identified as potential candidates based on this preliminary review. Next, a detailed analysis of the features of each and its primary use were conducted. A total of 5 key stakeholders including the physicians and directors associated with MUSC Center for Telehealth and SC Telehealth Alliance were consulted. It was understood that Adobe Connect, Skype, Cisco WebEx, and Cisco Jabber could be used to deliver video-based telemedicine, but they were not currently used extensively for doing so. On the basis of this feedback, the telemedicine tools selected for this research were (1) Doxy.me, (2) Vidyo, (3) VSee, and (4) Polycom.

#### Doxy.me

**Figure 1:**
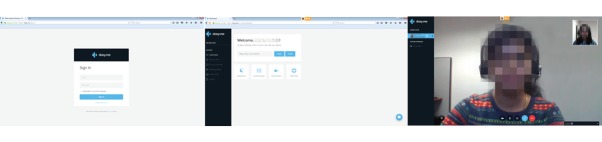
Doxy.me log-in screen, waiting room, and clinician’s view.

Doxy.me is a free Web-based system (as opposed to downloaded desktop application) specifically designed for telemedicine purposes. Clinicians create an account and a personalized waiting room where they communicate with their patients, either copying and emailing or directly emailing the address of their waiting room. By clicking on this link, patient is directed to the clinician’s waiting room. There is a self-view box at the top right and a chat box at the bottom right of the screen. Volume and video control buttons are located below the patient’s video. In addition to these features, the clinician can edit the waiting room and change the account settings. Figure 1 below shows the Doxy.me log-in screen, waiting room, and clinician’s view.

#### Vidyo

**Figure 2:**
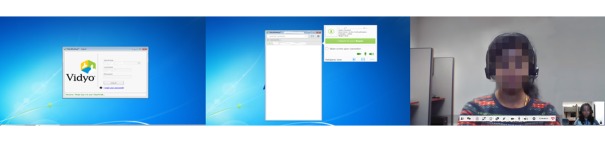
Vidyo log-in screen, contact list, and clinician’s view.

Vidyo is one of the leading telemedicine videoconferencing desktop-based application solutions. After creating an account, the clinician receives an email with log-in credentials and a Vidyo portal. The Vidyo desktop application is downloaded by clicking the portal. The clinician sends a Vidyo meeting invitation after logging into this application. He or she can change the video quality and other settings by clicking on the configuration button. In addition, this task bar includes the volume and video control buttons, group chat option, self-view option, screen layout option, and end call option. Figure 2 shows the Vidyo desktop application from the clinician’s perspective.

#### VSee

**Figure 3:**
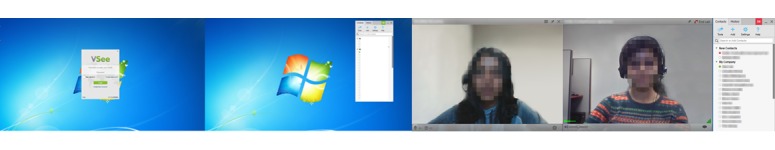
VSee log-in screen, application screen, and clinician’s view.

VSee is a telemedicine system which requires the clinician to create an account and install a desktop application. After logging into the account, the clinician invites patients by entering their email ids or copying and emailing them an invitation link. This system includes an option for text chatting with the patient as well as separate windows for self-view, clinician’s video, chat box, and contacts, with the microphone and camera settings being found on the self-view window. Figure 3 shows the VSee log-in screen, application screen, and clinician’s view.

#### Polycom

**Figure 4:**
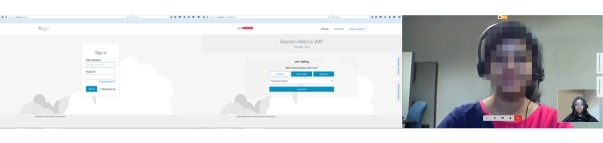
Polycom log-in screen, welcome screen, and clinician’s view.

Polycom, a licensed Web-based application that can be purchased from the Polycom website, provides telemedicine and video services for remote conferencing and collaboration. Although this company provides a hardware-based telemedicine solution, this study used a lightweight product for home-based care. The clinician receives an email with log-in credentials and a link for accessing the account. After logging into the account, the clinician selects the devices or system, which includes an option for adding participants and managing meetings. The clinician invites patients by emailing them the address of his or her chat room; after clicking this link, the patient is then directed to the meeting. There is a self-view option on the left side of the screen, the participant list on the right, and the patient’s video in the middle. The control buttons are located below the patient’s video. Figure 4 shows the Polycom log-in screen, welcome room, and clinician’s view.

### Study Personnel

The investigation reported here was based on the experts’ heuristic evaluations and the severity of the problems based on a severity rating scale. A heuristic evaluation is a discounted usability method in which the evaluation of an interface is done based on established usability principles. Five human factors engineers—three PhD students, one assistant professor, and one master’s degree student, all with prior training in conducting heuristic evaluations—were recruited to serve as the subject matter experts for this study. All received verbal information about the purpose and the goal of the study, and a detailed written task flow to guide the evaluation of the 4 telemedicine platforms using a modified heuristic evaluation procedure. They were compensated with a US $20 Amazon gift card for their time.

### Study Design and Procedure

The method of evaluation used in the study was a heuristic evaluation, a discounted usability evaluation method, combined with a severity rating scale [27,42]. Specifically, Nielsen’s heuristics were used because of their widespread use and acceptability [27,43-45]. The heuristics, which are listed in Table 1, were used to highlight possible usability issues.

**Table 1:** Usability heuristics used for evaluating the telemedicine interfaces (adapted from Nielsen’s heuristics [27,28]).

Heuristic	Description
Visibility of system status	The system should always keep users informed about what is going on, through appropriate feedback within a reasonable amount of time.
Match between system and the real world	The system should speak the users’ language, using familiar words, phrases, and concepts rather than system-oriented terms. Follow real-world conventions, making information appear in a natural and logical order.
User control and freedom	Users often choose system functions by mistake and will need a clearly marked “emergency exit” to leave the unwanted state without having to go through an extended dialog. Support undo and redo.
Consistency and standards	Users should not have to wonder whether different words, situations, or actions mean the same thing. Follow platform conventions.
Error prevention	Even better than good error messages is a careful design which prevents a problem from occurring in the first place. Either eliminate error-prone conditions or check for them and present users with a confirmation option before they commit to the action.
Recognition rather than recall	Minimize the user’s memory load by making objects, actions, and options visible. The user should not have to remember information from one part of the dialog to another. Instructions for use of the system should be visible or easily retrievable whenever appropriate.
Flexibility and efficiency of use	Accelerators—unseen by the novice user—may often speed up the interaction for the expert user such that the system can cater to both inexperienced and experienced users. Allow users to tailor frequent actions.
Esthetic and minimalist design	Dialogs should not contain information that is irrelevant or rarely needed. Every extra unit of information in a dialog competes with the relevant units of information and diminishes their relative visibility.
Help users recognize, diagnose, and recover from errors	Error messages should be expressed in plain language (no codes), precisely indicate the problem, and constructively suggest a solution.
Help and documentation	Even though it is better if the system can be used without documentation, it may be necessary to provide help and documentation. Any such information should be easy to search, focused on the user’s task, list concrete steps to be carried out, and not be too large.

A heuristic evaluation, typically conducted with 5 experts, detects up to 80% of the problems [18]. For this study, the experts individually conducted the assessment in a closed lab setting to avoid bystander bias. A 5-point severity scale was applied to each of the usability issues to indicate the level of concern [27]. The scale ranged from issues which may not impact the usability of the system to issues that could potentially lead to its failure. The 5-point scale is as follows [46]:

0—May not be a problem: other observers do not agree that this is a usability problem

1—Cosmetic problem only: it need not be fixed unless extra time is available

2—Minor usability problem: fixing it should be given low priority

3—Major usability problem: it is important to fix it, should be given high priority

4—Usability catastrophe: imperative that it is fixed before product can be released

Understanding the source of errors in a task begins with an in-depth understanding of the task flow [33]. This study, thus, began with a detailed task analysis for each of the 4 telemedicine systems to help understand the feedback they provide and the potential problems the user could face. This task analysis also included determining the knowledge the user must have in order to perform the task successfully. Before actual evaluation, the researcher discussed the detailed task analysis, heuristics, and severity ranking scale with the experts. As the experts were from the field of human factors and familiar with heuristic evaluation studies, only context-specific instructions were provided to evaluate the telemedicine platforms. A separate sheet containing the list of the heuristics and the severity rating chart was also given to the experts for their reference. The experts then evaluated the systems from the clinician’s perspective with the help of a hypothetical patient with whom no communication was carried out. After which, they listed the heuristic violations individually. The tasks to be completed by the evaluators were as follows:

Initiation: Create an account, log into the portal or desktop application, send an email invitation for the telemedicine session, call the patient.

Consultation: Toggle microphone and video, enter full screen mode, enter data into a chat box (where applicable), and end video call.

**Figure 5:**
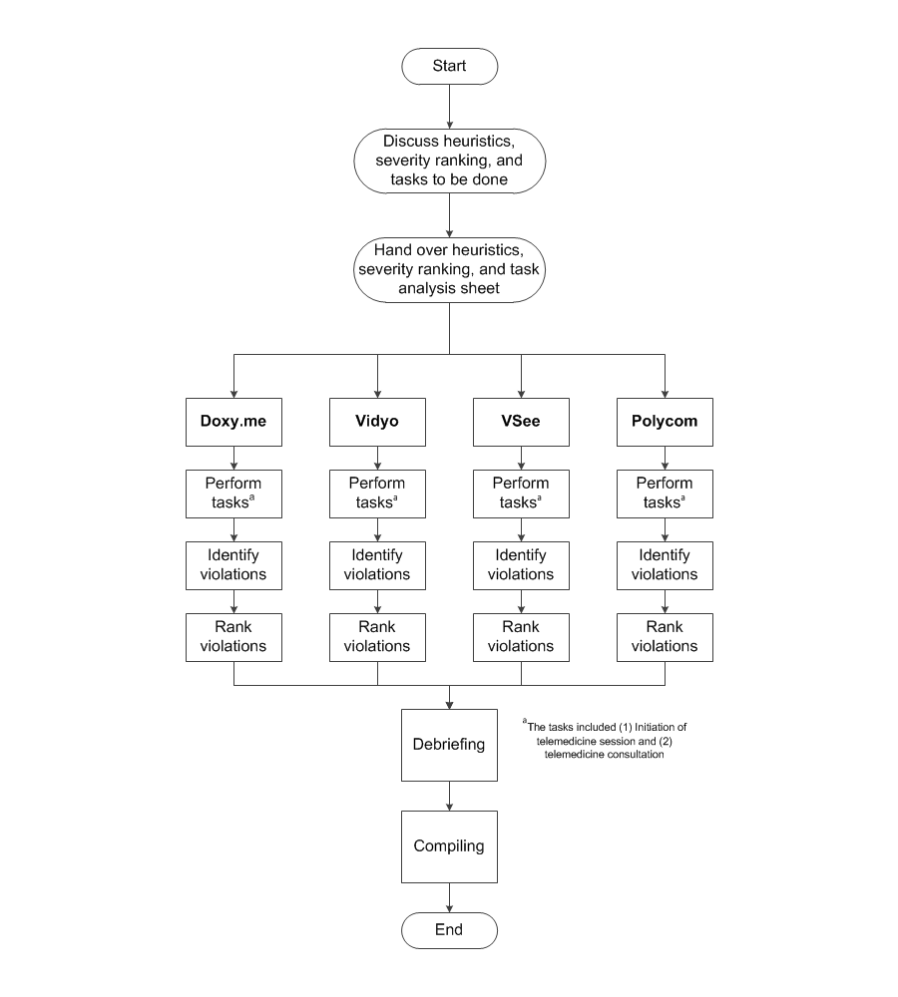
Experimental procedure followed.

On completing individual evaluations, experts discussed their findings with others in a postevaluation debriefing session. In the case of extreme inconsistencies, the evaluators discussed and came to a consensus about the appropriate rating. Individual lists were subsequently compiled for data analysis. Figure 5 outlines the experimental procedure followed in this study which lasted approximately 1 h.

### Data Analysis

For the evaluation, each expert recorded the heuristic violations for the respective tasks, including grading the severity of the issues. The individual ratings from the evaluators were averaged to obtain a single value of the severity. These data were then compiled to understand which heuristics were most violated and the severity was analyzed to prioritize the problems. The number of heuristics violated was graphed for the telemedicine session initiation and consultation.

## Results

### Heuristic Violations

**Figure 6:**
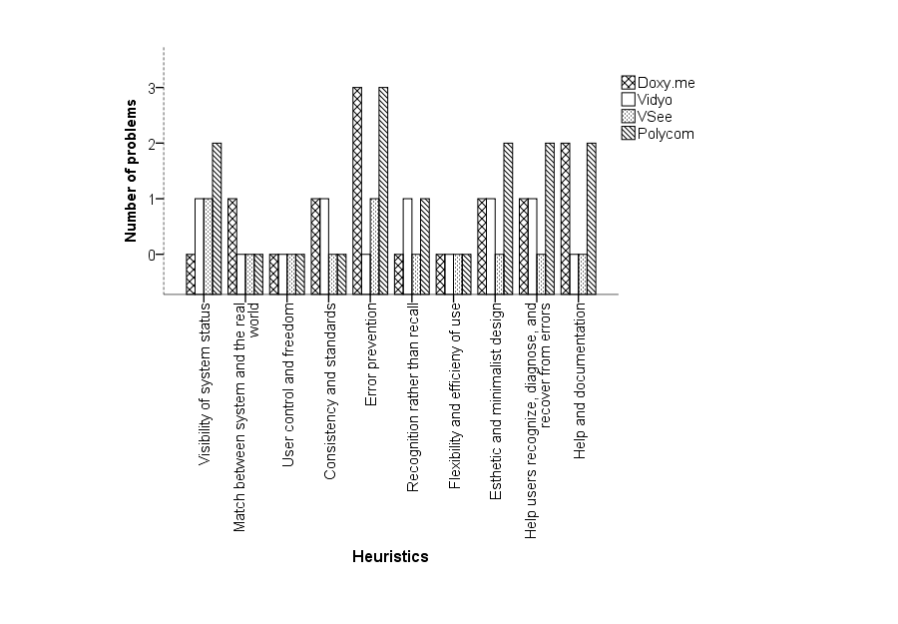
Heuristics violated during the telemedicine initiation session.

**Figure 7:**
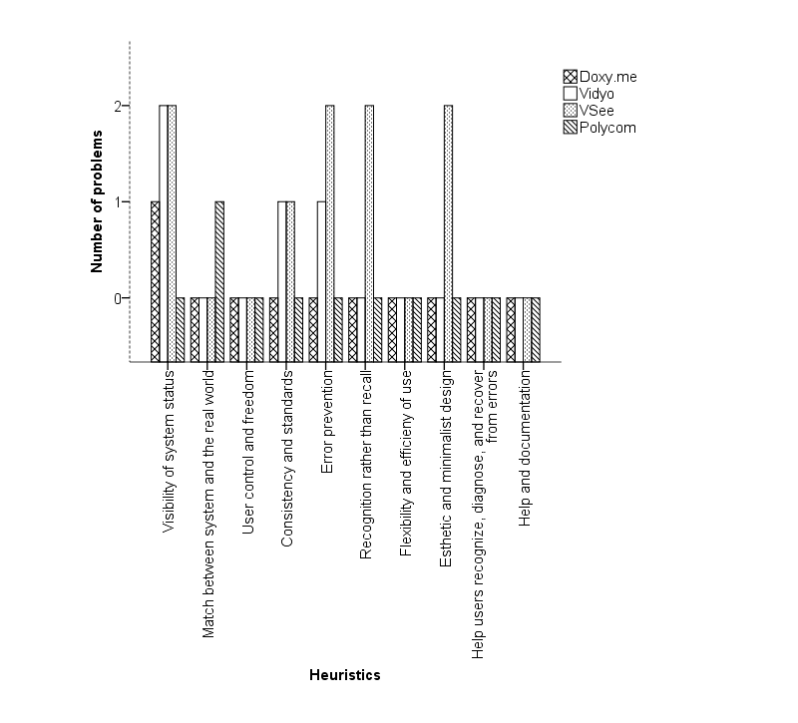
Heuristics violated during the telemedicine consultation.

**Figure 8:**
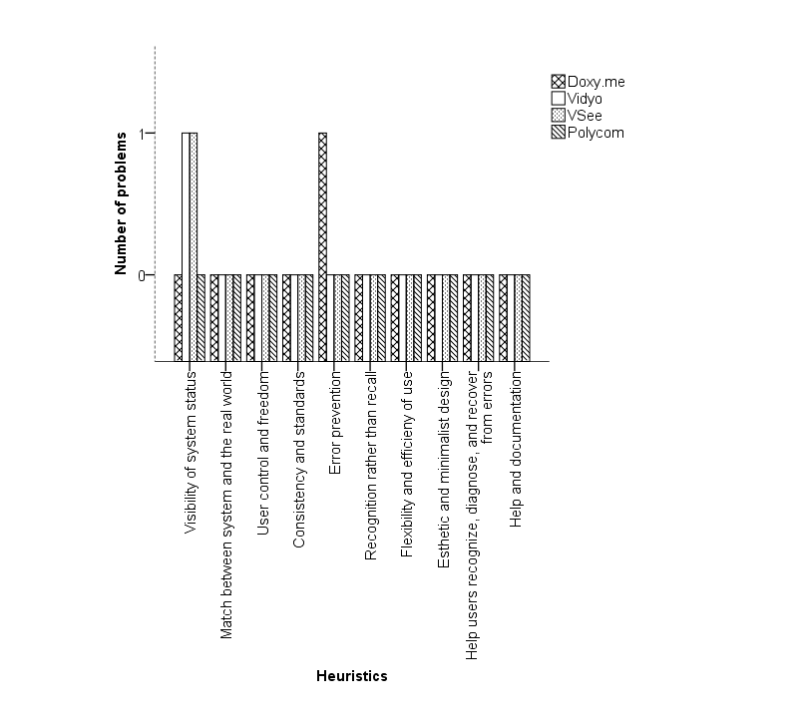
Heuristics violated during the telemedicine session conclusion.

**Figure 9:**
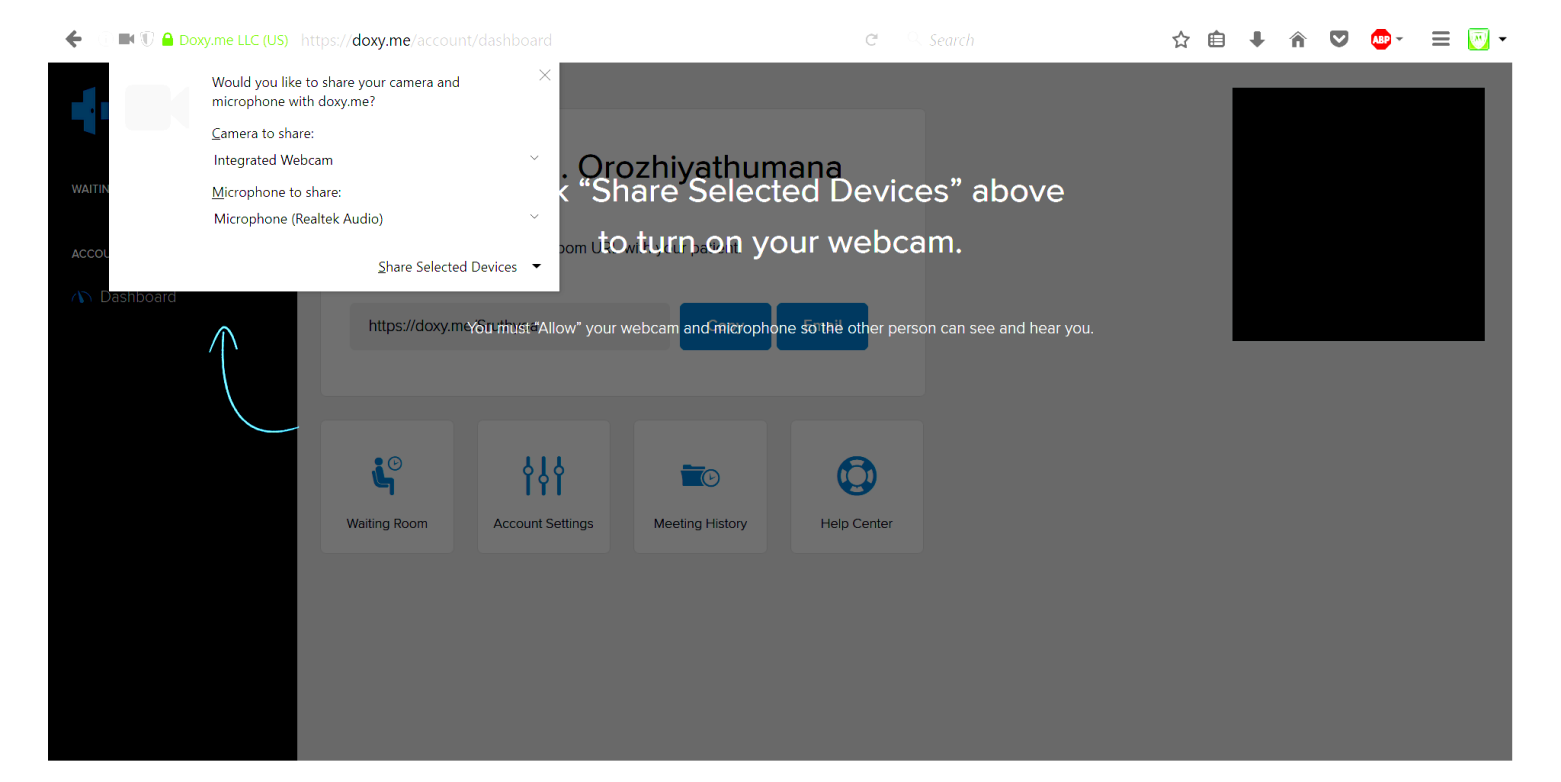
Microphone and camera sharing option.

**Figure 10:**
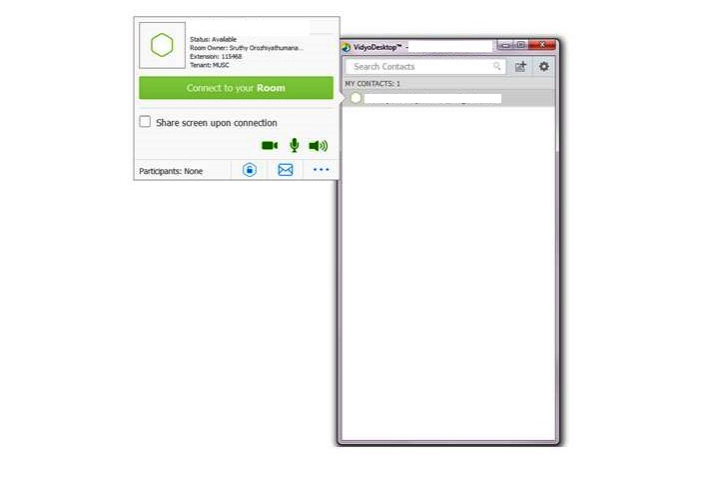
Absence of log-out option.

The heuristic evaluations of the experts for the clinician’s interface revealed a total of 46 unique issues: 11 for Doxy.me, 10 for Vidyo, 12 for VSee, and 13 for Polycom. Of these, 22% (10/46) concerns were recognized by all the experts. Tables 2-4 list the important usability issues and the heuristics they violated for initiating, conducting, and concluding a telemedicine session, respectively. Figures 6-8 are graphical representations of heuristic violations for initiating, conducting, and concluding a telemedicine session, respectively. As these figures show, 60.9% (28 issues) of the issues was identified in the initiation phase, 33% (15/46 issues) in the telemedicine session phase, and 7% (3/46 issues) in the conclusion phase. Sharing or setting up microphone and camera was one of the specific issues observed in the initiation phase (Figure 9). Using default email client (Microsoft Outlook) to invite patients was one of the issues identified during the telemedicine session. During conclusion, difficulty to find the log-out button was pointed out as an important issue (Figure 10).

**Table 2:** Heuristic violations identified in the telemedicine initiation session.

Task	Problem description	Solution recommendation	Heuristic violated	Severity rating
**Doxy.me**				
Entering room name (Doxy.me)	User may not comprehend the meaning of “room name” in a Web-based system setting	Provide explanation or rename as “clinician’s name”	Match between system and the real world	2.5
Select check boxes	The check boxes are not noticeable	Make the check box more noticeable	Error prevention	4
Sharing camera and microphone	Instruction says “allow” camera and microphone, but popup says “share”	Keep the instruction consistent with the website features	Consistency and standards	3
An email verification is sent to the user	Email is very lengthy and has too many links	Email could consist of a body with just the essential links	Esthetic and minimalistic design	3.5
**Vidyo**				
Click on the link in the welcome email sent by the company	Clicking on the link does not lead to log-in page. Instead, it downloads the application	Reword the link description or the link must lead to the log-in page	Consistency and standards	0
Click on email icon on top right corner of the application	Not all users may recognize the icon of an envelope as symbolizing email	There could be a label below the icon	Error prevention	3
Click log-in	Log-in button remains inactive if the portal is entered after entering username and password	Application should show an error message	Help users recognize, diagnose, and recover from errors	3
**VSee**				
Downloading .exe file	Does not prompt to confirm if download should be initiated. Download starts automatically	Notify user before downloading. Provide a prompt asking the users if they want to download the application at that moment or later	Visibility of system status	3.5
Enter email address in “Enter your email” tab	Email id is mandatory and cannot proceed to free video sign up option	Email entry could be on top with the sign-up for free tab below it instead of beside it	Error prevention	3
**Polycom**				
Click on the your account URL in the welcome email	Email contains a link for online account and another link for downloading. User might get confused and download application	Provide sufficient information about each link	Error prevention. Help and documentation	4
Selecting check in devices and software	There is multiple check in options which may confuse users	Provide sufficient information about each device and software	Error prevention. Help and documentation	3
Set camera, microphone, and speaker	There is no option to go back and redo these actions if they are missed	Provide an option to do these checks when necessary	Help users recognize, diagnose, and recover from errors	2

**Table 3:** Heuristic violations identified during the telemedicine session.

Task	Problem description	Solution recommendation	Heuristic violated	Severity rating
**Doxy.me**				
Click on email tab on the right side of the link	Not enough information provided about copy and email tabs. Directly going to default mail client	Popup boxes explaining meaning or function. Users need to choose the email client	User control and freedom	3.3
Enter data in chat box provided at the bottom right corner of the screen	The notification for an incoming message is not salient. Only the chat box symbol turns red	Make the chat box header turn another color	Visibility of system status	4
**Vidyo**				
Click on email icon on top right corner of the application	Clicking on the icon directly leads to default mail client	Application needs to allow user to choose preferred email client	User control and freedom	4
Click on your name	Room owner’s name is shown under “my contacts.” Tab or option to the chat room is not obvious	Popup with “connect your room” button needs to pop up when the user hovers the mouse over the name	Visibility of system status	3.3
Patient comes online	No popup when the patient comes online	Popup and audio notification to indicate that the patient is online	Visibility of system status	3
**VSee**				
Inviting patient	Direct email invitation and copy option are next to each other. This may confuse the user about whether both actions must be completed or only one	A simple “or” in between the two tabs	Esthetic and minimalist design	3
Disabling video and audio	Video and audio toggling icons are not salient	Icon could be presented in a brighter color	Recognition rather than recall	3
Enlarging the screen	Application does not have a full screen button. User has to drag to enlarge the screen	Provide a full screen button	Consistency and standards	4
**Polycom**				
Compose email in default email client	The invitation to the patient has to be sent from the default email client	Application must provide the option to the user regarding preferred email client	User control and freedom	4
Compose invitation email to patient	Application always redirects to default mailer	An option to enter patient’s email id could be provided with an example invitation email	User control and freedom	4
Compose email in default email client	Directly goes to default mail client	Allow user to choose preferred email client	User control and freedom	4
Enter full screen by clicking on full screen icon at the bottom of the page	System says “my rpcloud.vc” is full screen when the view is changed to full screen mode	Use terms which user can easily understand	Match between system and the real world	1

The heuristics most frequently seen violated were *visibility of system status* and *error prevention,* each with 11 violations (24%, 11/46), with *esthetic and minimalist design* being second with 6 out of 46 violations (13%, 6/46). Violations were not observed for the heuristics *user control and freedom* and *flexibility and efficiency of use*. It was found that (1) 4 out of 46 (9%, 4/46) violations were recorded for each of *consistency and standards, recognition rather than recall, help users recognize, diagnose, and recover from errors* and *help and documentation* heuristics, and (2) 2 out of 46 (4%, 2/46) violations were observed for *match between system and real world* heuristic. Specific issues related to *visibility of system status* included lack of feedback while downloading setup (.exe), lack of saliency of notifications on receiving a message or when a patient enters a Web-based waiting room, and the absence of salient call end and log-out icons. Inconspicuous check boxes, inadequate labeling of icons, and failure to exit full screen on completion of a session were identified under *error prevention*.

**Table 4:** Heuristic violations identified while concluding the telemedicine session.

Task	Problem description	Solution recommendation	Heuristic violated	Severity rating
**Doxy.me**				
Click on red phone icon at the bottom of the page to end session	Remains in full screen even after ending the call	A dialog box saying press escape or automatically escape from full screen	Error prevention	1
**Vidyo**				
Log-out of the application	No obvious log-out button	Provide conspicuous log-out button	Visibility of system status	4
**VSee**				
Click on log-out	Log-out button is not easily seen	Provide conspicuous log-out button	Visibility of system status	3
**Polycom**				
No issues were identified				

### Issues Requiring Immediate Attention

**Figure 11:**
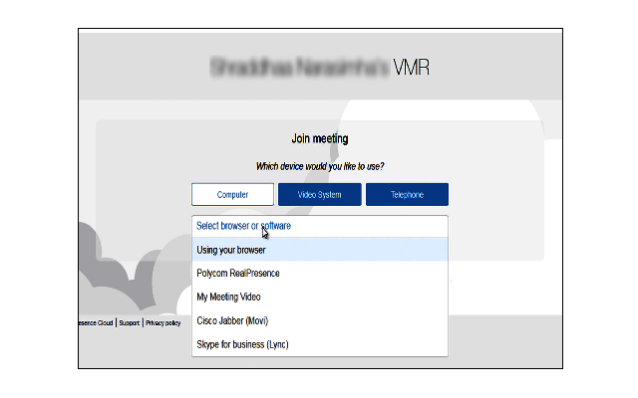
Multiple log-in options in Polycom.

The experts rated two issues as requiring immediate action. The multiple check-in options available in the welcome screen of Polycom was one, with the experts finding that the availability of multiple options confused the user, and that the welcome screen did not contain sufficient information to choose the appropriate device (Figure 11). The second problem highlighted by the experts was the use of a default email client to email invitations to the patients. Three (Doxy.me, Vidyo, and Polycom) of the four conditions redirect the user to the default email client to send email invitations to the patients. It may be more effective to give the user the choice of using the email client of his or her choice.

During the debriefing, experts discussed their most and least favorite aspects of each of the platforms. Experts indicated that they preferred interfaces that were not cluttered with too many options, language that they could relate to that in the real world, and systems that provided adequate and timely feedback for their actions. The least enjoyable aspects were welcome emails from the telemedicine platforms with multiple links, the use of a default email client to invite patients, and the failure of many options to respond the way expected.

## Discussion

### Principal Findings

The heuristic evaluation was conducted using a structured table containing the task flow and a column for experts to input the problems they found for each task, the respective heuristic violated, the severity of the violation, and possible solutions. Several issues were identified during the course of this evaluation. Visibility, error prevention, and minimalistic design issues were frequently violated. The effect of such issues on the ability of a user to process information can be explained using the information processing model [47]. A detailed description of the different aspects of the information processing model that are affected due to these issues would result in developing better solutions.

**Figure 12:**
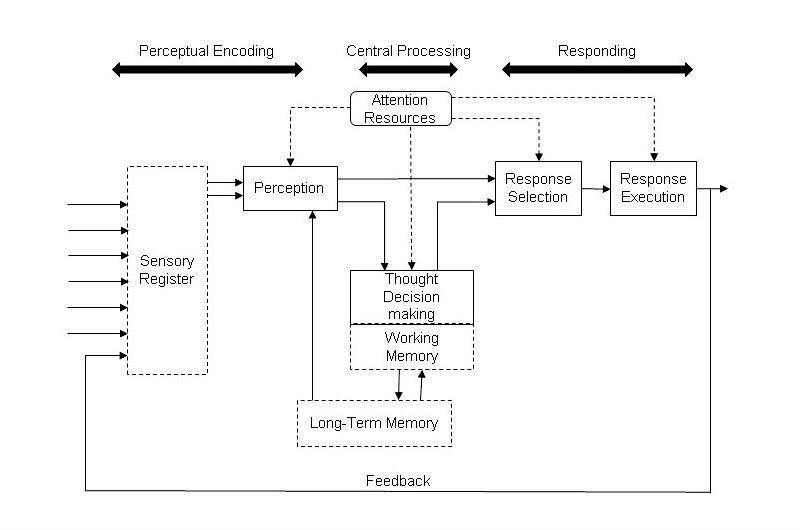
Information processing model.

The information processing model [47] can be used to understand the impact of these issues on the user’s ability to understand and make decisions (Figure 12). This model illustrates the procedure of the human cognition process. The sensory register includes our sense organs that help a person take cues from our surroundings, which then leads to understanding or perceiving these cues. The working memory refers to the understanding and retention of information only for the span of completing a task. However, the long-term memory involves the retention of information for longer period of time such as a few weeks, months, or years. Using the information in the working memory and the long-term memory, the process of thinking and decision-making occurs to make a decision about the cues obtained from the environment. Once thoughts about the cues have been formed, an appropriate response to the cue is developed and based on this, an action is executed in response to the cue obtained. The execution of the response is again taken in by the sensory register and stored in the long-term memory for future situations. Throughout this process, there is also a constant requirement of attentional resources which help the user to focus on the necessary information and eliminate the rest.

On the basis of the issues specified, the lack of feedback would have a direct effect on the perception of the process. As a result, the user would have difficulty in deciding the subsequent process to be carried out in the procedure. It was also indicated that the popup for the chat box was not salient. This would directly affect the sensory register as the popup would not be visible and hence the user would fail to understand (perception) that a message has been received. The icon size and design would again affect sensory register and perception. The content of the email invitation, which was reported to require immediate attention, will prove to be an important issue affecting the working memory. The working memory, responsible for understanding and retaining information until the completion of a task, would be affected due to the large amount of information or the lack of information in the email invitation. Another problem reported as requiring immediate attention was the use of default email client to send email invitations to patients. This could potentially require the retrieval of passwords to log into the system which affects the long-term memory.

Although of lesser importance, there exist some other issues which must be studied with respect to their impact on a user’s decision-making. One such issue is the need to enter large amount of data for registration. This could affect the working memory limits of a person as they would be required to read and retain multiple data to enter. In three of the four platforms analyzed—Doxy.me, Polycom, and Vidyo—it was seen that the clinician was required to send an email invitation for every meeting. This would add to the working memory limits to process immediately available information and the long-term memory to remember patient name and email address to send the emails. The popups used to share the microphone and camera was indicated to be inconspicuous resulting in additional load on the sensory register due to lack of visibility.

On the basis of the understanding of the different areas of the information processing model affected by the issues and the issues highlighted by the experts, certain design recommendations were developed for telemedicine systems. Some of the key findings for improving the interaction of physician with the interface to enhance the usability of telemedicine platforms are given in Textbox 1.

**Key findings for improving the physician-interface interaction.**

Telemedicine initiation

- Provide browser-based applications rather than desktop applications. VSee and Vidyo require their respective applications to be installed on the user’s device. A browser-based application avoids the installation process
- Highlight required field in the registration process and provide clear error message when the users fail to fill it
- Ask for only limited information for creating an account and provide an option to update user profile later
- Send an easy-to-comprehend and simple welcome email to the users upon account creation. The link which connects to the telemedicine platform needs to be highlighted as a hyperlink or a button
- Include an introductory tutorial to help users understand the different options the platform offers. Only VSee provides a tutorial tour upon logging into the application for the first time. Most of the tutorial tours do not appear as soon as the users log into the platform for the first time. They need to find the option (hidden in most cases) to watch the tour
- Give users the freedom to choose their email client without connecting it to the system’s default. Vidyo directly links to the default email client. If the clinician does not have an account with it, he/ or she cannot send an invitation. Polycom and Doxy.me allow users to choose among a number of email clients. Doxy.me and VSee allow users to log in to their email externally and send an invitation to a patient by copying and pasting the URL
- Have an option to save added contacts so that the user can contact patients again without having to go through the process of inviting them. Currently, only 1 of the 4 telemedicine platforms, VSee, has this feature

Telemedicine consultation

- Provide options for users to check their microphone and speaker connectivity before every conversation. Polycom has a foolproof system that allows users to join the conversation only if the connections are working. VSee allows the user to set up audio and video during the first log-in attempt. For the other two platforms, the clinician does not realize issues with the connectivity until he begins the conversation. Doxy.me instructs the user to click “allow” to share devices. However, the popup that appears does not have an allow button
- Provide adequate and clear feedback when major tasks like adding a patient, accepting a patient, and ending call are performed. When patients accept the invitation on Vidyo, it gives auditory notification; however, there is no popup notification of their status. For Polycom, only the number 1 appears on the side of screen; however, that number does not signify anything to the clinician. Doxy.me sounds a chime, and the patient’s name appear on the side; however, it is hard to notice because it usually blends into the page
- Provide conspicuous icons with popup feedback. VSee and Vidyo do not have salient log-out buttons, making the log-out process difficult
- Make the interface simple. Most frequently used icons can be made static, labeled, and grouped together. Cluttered interface with multiple windows and scattered icons may confuse users

or Apart from providing recommendations for improvement based on the information processing model, this study also demonstrates the practicality and ease of applying heuristic evaluation in usability studies. The entire process of conducting the study and analyzing the results took a week’s time and did not involve the use of any software applications. The efficiency of this method makes it well-suited for use during the early development stages [18].

### Limitations

This study is not without its limitations. In this study, we evaluated the telemedicine systems on a Windows 7 computer with Mozilla Firefox browser only. These systems need to be tested on multiple operating systems and Web browsers. Also, the evaluators for this study were not medically trained professionals. Future studies have to be conducted with actual clinicians to find usability issues from their perspective. Furthermore, as indicated by Nielsen and Molich [27], this heuristic evaluation like all others, only helps to identify the usability issues without providing solutions to address them.

### Conclusions

Multiple studies have been carried out explaining the effectiveness of telemedicine in providing medical care with little research focusing on the ease and usability of these systems [24]. In this study, we used a heuristic evaluation and severity rating method to assess the usability of 4 telemedicine software platforms with a focus on understanding the interface issues faced by the clinician. Furthermore, the information processing model was used as the baseline to explain the impact of these issues on the user’s capability in making decisions. The heuristic evaluation and severity rating method was found to be effective in uncovering issues in the interface as 46 unique issues were uncovered across 4 different platforms. Prominent issues among these, whose impact was explained using the information processing model, is an indication of the need for further human factors concept-based studies of the interfaces of telemedicine systems.

With a focus on the clinician’s interface design, this heuristic evaluation was found to be an effective method for uncovering violations. This heuristic evaluation identified only potential usability problems in an existing interface; usability studies involving physicians could further indicate aspects of the system that work well and identify the most appropriate functionalities. However, with limited resources available, heuristic evaluation is a practical, affordable, and efficient method for revealing usability problems. Experts liked systems that had a straightforward and simple interface and that did not require installation. In addition, they preferred systems that sent simple welcome emails. From a telemedicine point of view, this is important as clinicians and technicians do not have the time to spend navigating and comprehending complex platforms.

Heuristic evaluation is a discounted usability evaluation method with limited generalizability. Future studies need to focus on detailed usability evaluation with actual clinicians and patients. Conducting retrospective interviews with the users help the designers understand their needs and in turn design or modify the system appropriately.

## Abbreviations

HIPAA: Health Insurance Portability and Accountability Act
IOM: Institute of Medicine
MUSC: Medical University of South Carolina
SC: South Carolina
